# Supplementary figures and images for: PolarMorphism enables discovery of shared genetic variants across multiple traits from GWAS summary statistics
Source: Bioinformatics. 2022 Jun 27;38(Suppl 1):i212–9. doi: 10.1093/bioinformatics/btac228 (PMC9235478; doi:10.1093/bioinformatics/btac228)

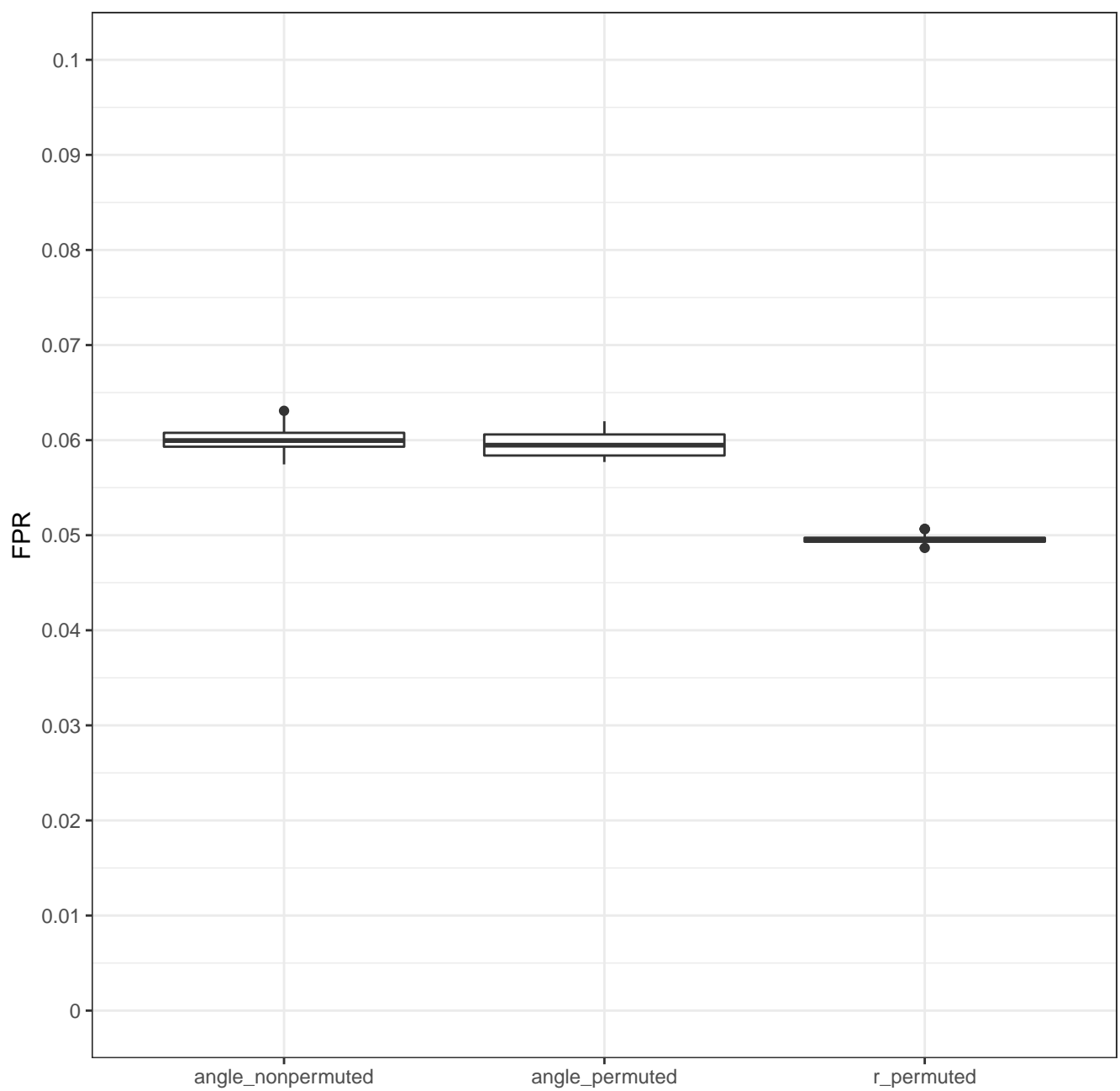

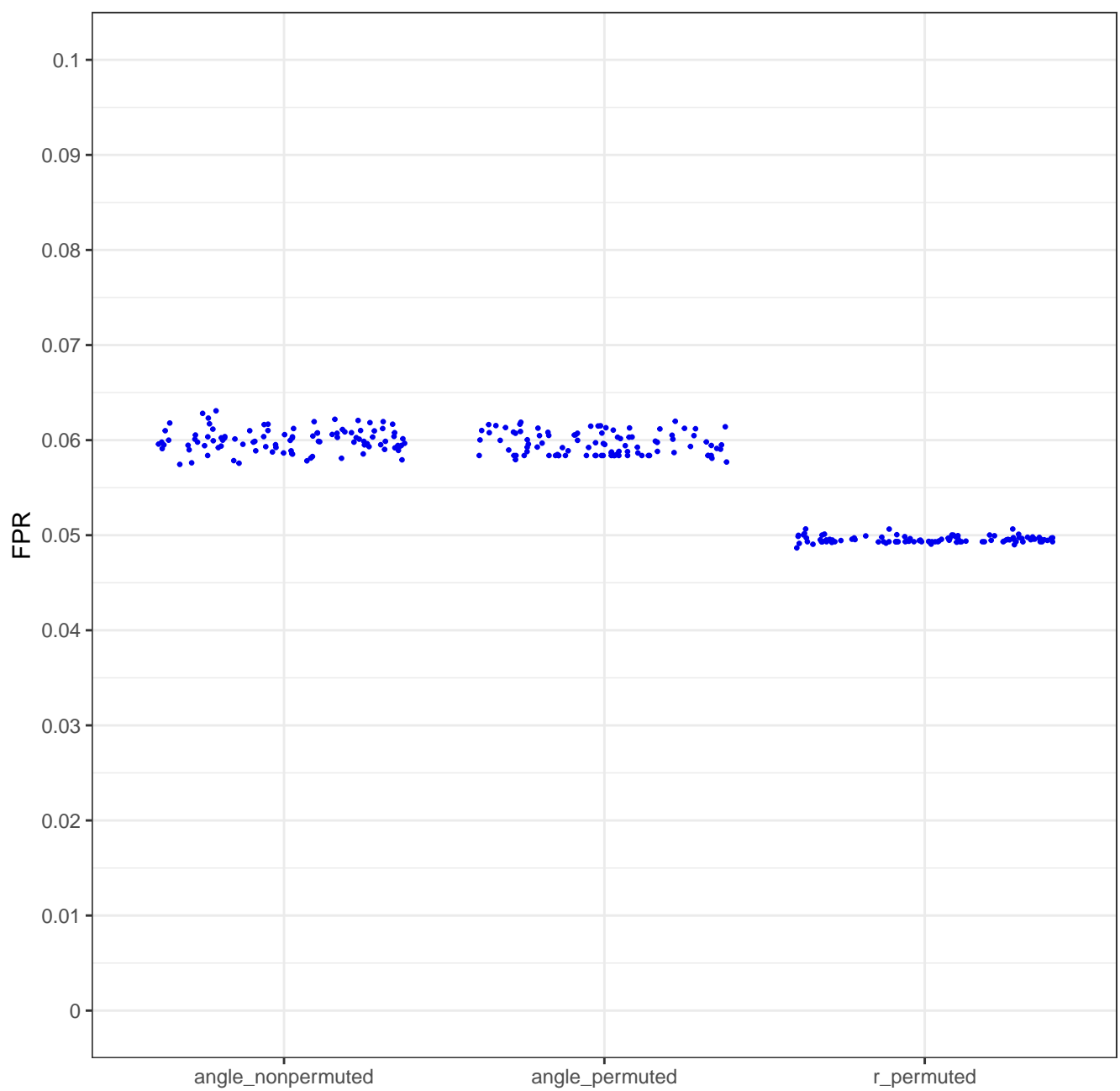

Supplement: btac228_Supplementary_Data [file btac228_supplementary_data.zip › btac228-Suppl_data/vonBerg.235.sup.fig.1.pdf]

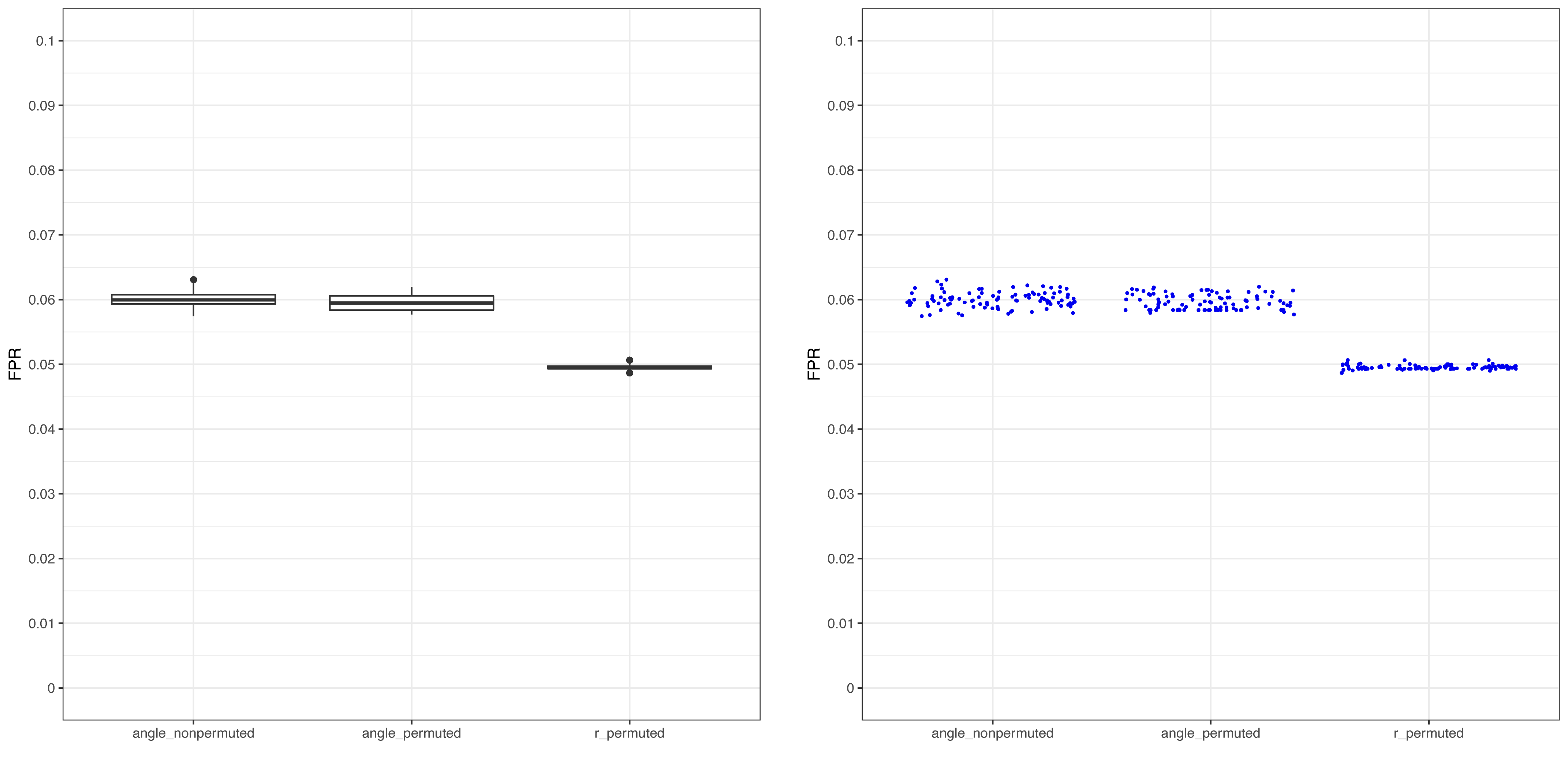

Supplement: btac228_Supplementary_Data [file btac228_supplementary_data.zip › btac228-Suppl_data/vonBerg.235.sup.fig.1.png]
